# Cross-talk between the Tissue Factor/coagulation factor VIIa complex and the tyrosine kinase receptor EphA2 in cancer

**DOI:** 10.1186/s12885-016-2375-1

**Published:** 2016-05-31

**Authors:** Oskar Eriksson, Åsa Thulin, Anna Asplund, Geeta Hegde, Sanjay Navani, Agneta Siegbahn

**Affiliations:** Department of Medical Sciences, Clinical Chemistry & Science for Life Laboratory, Uppsala University, Uppsala, Sweden; Department of Immunology, Genetics & Pathology & Science for Life Laboratory, Uppsala University, Uppsala, Sweden; Lab Surgpath, The Human Protein Atlas Project, Mumbai Site, Mumbai, India

**Keywords:** Tissue Factor, Coagulation factor, EphA2, Colorectal cancer, Cell signaling

## Abstract

**Background:**

Tissue Factor (TF) forms a proteolytically active complex together with coagulation factor VIIa (FVIIa) and functions as the trigger of blood coagulation or alternatively activates cell signaling. We recently described that EphA2 of the Eph tyrosine kinase receptor family is cleaved directly by the TF/FVIIa complex. The aim of the present study was to further characterize the cross-talk between TF/FVIIa and EphA2 using in vitro model systems and human cancer specimens.

**Methods:**

Cleavage and phosphorylation of EphA2 was studied by Western blot. Subcellular localization of TF and EphA2 was investigated by a proximity ligation assay and confocal microscopy. Phalloidin staining of the actin cytoskeleton was used to study cell rounding and retraction fiber formation. Expression of TF and EphA2 in human colorectal cancer specimens was examined by immunohistochemistry.

**Results:**

TF and EphA2 co-localized constitutively in MDA-MB-231 cells, and addition of FVIIa resulted in cleavage of EphA2 by a PAR2-independent mechanism. Overexpression of TF in U251 glioblastoma cells lead to co-localization with EphA2 at the leading edge and FVIIa-dependent cleavage of EphA2. FVIIa potentiated ephrin-A1-induced cell rounding and retraction fiber formation in MDA-MB-231 cells through a RhoA/ROCK-dependent pathway that did not require PAR2-activation. TF and EphA2 were expressed in colorectal cancer specimens, and were significantly correlated.

**Conclusions:**

These results suggest that TF/FVIIa-EphA2 cross-talk might potentiate ligand-dependent EphA2 signaling in human cancers, and provide initial evidence that it is possible for this interaction to occur in vivo.

**Electronic supplementary material:**

The online version of this article (doi:10.1186/s12885-016-2375-1) contains supplementary material, which is available to authorized users.

## Background

The Eph receptors are the largest family of receptor tyrosine kinases (RTKs) in humans with 14 members. Eph receptors are activated by cell–bound ephrin ligands, and the Eph-ephrin system governs contact-dependent intercellular communication controlling a wide array of biological processes such as development, tissue organization and cell migration [1, 2]. EphA2 of the A type Eph subclass is expressed at low levels in differentiated tissues but expression frequently increases in advanced cancers, implicating EphA2 in tumor progression [3]. The preferred ligand for EphA2 is ephrin-A1 [4], and ligation of EphA2 by ephrin-A1 leads to the formation of multimeric receptor-ligand clusters that activate a signaling response that controls cytoskeletal dynamics and cell morphology. While ligand-dependent EphA2-activation has been considered tumor suppressive, recent reports have highlighted a role for EphA2-ephrin-A1 signaling in tumor cell plasticity and a shift from mesenchymal to amoeboid morphology [5, 6] and increased single cell invasion [7]. In addition, oncogenic EphA2 signaling has been proposed to be ligand-independent, drawing from the observations of decreased expression of the ephrin-A1 ligand paralleling increased EphA2 expression in human cancers [8]. Miao et al. showed that EphA2 is a substrate and effector of PI3 kinase/Akt signaling through phosphorylation of serine 897 in the EphA2 cytoplasmic domain, a pathway by which EphA2 controls cancer cell motility and invasion independently of ephrin-A1 [9, 10].

Tissue Factor (TF) is the receptor and co-factor for coagulation factor VII/VIIa (FVII/FVIIa), a circulating serine protease. The proteolytic TF/FVIIa complex functions as the physiological trigger of blood coagulation and in addition activates cell signaling through mechanisms dependent or independent of protease-activated receptors (PARs) and the TF cytoplasmic domain [11]. TF expression is found in tumor cells [12], and in preclinical models, TF/FVIIa signaling has been implicated in tumor progression through effects on processes such as cell migration and angiogenesis [13, 14]. Furthermore, a clinically relevant role of the coagulation system in malignancies is evidenced by the increased risk of thrombosis in cancer patients. In contrast, anticoagulant treatment only modestly influences cancer incidence and survival in humans, and the effect seem to differ between cancer types [15].

We previously reported on a direct cleavage by TF/FVIIa in the ligand binding domains (LBD) of the Eph receptors EphB2 and EphA2. We also identified a conserved disulfide bond that kept the N-terminal fragment tethered to the receptors after cleavage [16]. In this study we set out to further explore how TF/FVIIa influences EphA2 signaling and activity. We report herein that TF and EphA2 co-localizes in MDA-MB-231 breast cancer cells with constitutive high TF expression and in TF transfected U251 glioblastoma cells, and that FVIIa sensitizes MDA-MB-231 cells to ephrin-A1-mediated cytoskeletal reorganization and cell rounding independently of PAR2-activation through a RhoA/ROCK pathway. EphA2 and TF were co-expressed in a cohort of human colorectal cancer specimens, providing evidence that the prerequisites for TF–EphA2 cross-talk in vivo are present.

## Methods

### Reagents

Antibodies towards EphA2 (6997), pS897-EphA2 (6347), pY588-EphA2 (12677) and GAPDH (2118) were from Cell Signaling Technology. The RhoA antibody (ARH04) was from Cytoskeleton. The TF antibody (clone 10H10) was a kind gift from Professor J Morrissey (University of Illinois) and the PAR2 blocking antibody was a kind gift from Professor W Ruf (Scripps Institute). PI3 kinase inhibitor LY294002 was from Calbiochem and ROCK inhibitor Y-27632 from Sigma.

### Cell culture

MDA-MB-231 cells were obtained from the American Type Culture Collection and cultured in complete RPMI 1640 medium. For experiments, cells were seeded in individual wells and left to attach over night. Cells were then switched to medium containing 0.1 % FBS, for the FVIIa groups supplemented with 10 nM FVIIa for 1 h and then stimulated with ephrin-A1 or Fc control as indicated in the figure legends. Prior to experiments, ephrin-A1 was preclustered for 1 h at room temperature with anti-human Fc goat IgG (Jackson ImmunoResearch) at 1:10 concentration. Preclustered Fc fragments (Jackson Immunoresearch) were used as controls. In some cases, cells were pretreated for 30 min with inhibitors prior to stimulations.

U251 cells were from Cell Line Services and were cultured in complete DMEM medium. Experiments were performed in DMEM with 0.1 % FBS.

### SDS-PAGE and Western blot

SDS-PAGE and Western blot was performed using the Novex Bis-Tris gel system (Life Technologies) as previously described [14].

### mRNA analyses

Total RNA was extracted from cells by Trizol® (Life Technologies) using standard protocols, and converted to cDNA using oligoDT primers. Quantitative real-time PCR (qPCR) was performed using Assays on demand (Applied Biosystems) for IL8 with β2-microglobulin as housekeeping gene on an ABI prism 7500 system. Results were calculated using the comparative CT method for separate tubes.

### siRNA knock-down of RhoA

MDA-MB-231 cells were transfected with 10 nM RhoA siRNA (Silencer select, Ambion) using Lipofectamine RNAiMAX (Life Technologies) according to instructions from the manufacturer. Cells were assayed 48 h after transfection. Efficiency of protein knock-down was analyzed by Western blot.

### In situ proximity ligation assay

The assay was performed with reagents supplied by the manufacturer (Olink Bioscience) and according to instructions supplied with the kit. In brief, MDA-MB-231 cells were grown on chamber slides (Lab Tek), fixed in 4 % PFA/PBS, blocked and incubated with antibodies towards TF and EphA2, which were bound by secondary antibodies connected to specific oligonucelotides that ligate when they are in close proximity. Ligated oligonucleotides then serve as template for a rolling-circle amplification and the amplification products were visualized by fluorescently labeled probes. Cell nuclei were stained with DAPI and images captured with a 40x objective using a Zeiss Axioimager fluorescence microscope.

### Confocal microscopy

For microscopy experiments, cells were grown on 8-well chamber slides (Lab Tek), and then washed with PBS and fixed in 4 % PFA. Cells were permeabilized with 0.2 % Triton X-100 and subsequently blocked in 2 % BSA for 30 min. Antibody incubations were performed for 1 h at room temperature for primary antibodies (EphA2 1:200, TF 1:500) and 30 min in the dark at room temperature for secondary antibodies (1:1000) (Molecular Probes). Staining of the actin cytoskeleton with phalloidin-FITC (Sigma) diluted 1:500 was performed together with the secondary antibodies. Slides were washed with PBS between all steps, and mounted in Vectashield mounting medium with DAPI and sealed with nail varnish. Confocal images were captured with a Zeiss LSM710 confocal microscope using the 40x or 63x objectives.

### Assay for cell rounding and retraction fiber formation

MDA-MB-231 cells were seeded on 8-well chamber slides (Lab-Tek) coated with 10 μg/ml collagen IV (Sigma) and left to attach over night. After treatments, cells were washed with PBS and fixed in 4 % PFA/PBS. The cytoskeleton was stained by FITC-conjugated phalloidin (Sigma) for 30 min at room temperature protected from light. Slides were then washed, mounted in mounting media with DAPI and sealed with nail varnish. Images were captured using an Axiovert 40 CFL inverted epifluorescence microscope (Zeiss) and the 40x objective. 3–4 images per well were taken at random locations and the percentage of cells with rounded morphology and retraction fibers was quantified according to Taddei et al. [17].

### TF overexpression

U251 cells were transfected to transiently overexpress TF by using Lipofectamine 3000 (Life Technologies) and a plasmid encoding untagged human TF (Origene). Briefly, cells were seeded in 24-well plates, left to attach over night and transfected with 400 ng DNA. Lipofectamine without DNA was used as control, and stimulations were performed 24 h post transfection in low serum DMEM.

### Immunohistochemistry (IHC) of colorectal cancer specimens

The patient cohort used in the study contained non-consecutive cases diagnosed with colorectal cancer between 1990 and 2003 in the Uppsala region in Sweden, collected with the purpose to screen for protein expression differences between disease stages and between normal colorectal tissue, cancer and metastases. The cohort included 60 patients, with 20 (33 %) patients each in stages I, II and III. 41 (68 %) cases were colon cancers and 19 (32 %) cases were rectal cancers. 10 cases each of colorectal normal tissues and adenomas as well as 20 cases of lymph node or distant metastases were also included. Tumor and patient characteristics were based on the original histopathology reports and the patients’ clinical records.

All tumor material was present as formalin-fixed paraffin-embedded tissue in duplicate cores on tissue microarrays (TMAs), constructed at the SciLife Laboratory Tissue Profiling Facility at Uppsala University as described previously [18]. Expression of TF and EphA2 was detected by IHC using a rabbit polyclonal TF antibody developed by the Human Protein Atlas project [19] and a rabbit monoclonal EphA2 antibody (6997, Cell Signaling Technology). Automated IHC staining were performed as previously described [20], using a LabVision Autostainer 480S (ThermoFisher Scientific). Microarray sections were baked over night, deparaffinized, hydrated in graded alcohols and blocked for endogenous peroxidase using 0.3 % hydrogen peroxide. Following antigen retrieval, sections were stained with primary antibody (30 min) and secondary dextran polymer visualization system (30 min), followed by the addition of diaminobenzidine as chromogen. All solutions except primary antibodies were obtained from Laboratory Vision (Laboratory Vision). Sections were counterstained in Mayer’s hematoxylin (Histolab), before dehydration and mounting of coverslip. Stained slides were digitalized by scanning, using an Aperio ScanScope XT Slide Scanner (Aperio Technologies). Tumor cell staining was annotated semi-quantitatively with respect to staining intensity and fraction of positive cells. Intensity was graded as negative, weak, moderate or strong, and graded in six fractions (0–1 %; 2–10 %; 11–25 %; 26–50 %, 51–75 %; >75 %) and specimens with moderate or strong staining in more than 2 % of tumor cells were considered positive.

During TMA sectioning 6 primary cancer cases and 3 metastases were lost or compromised, resulting in TMA cores with no representative tumor tissue present. These cases were not annotated and excluded from all statistical analyses.

### Statistics

Statistics were performed using the GraphPad Prism (GraphPad Software) and Statistica (Statsoft Scandinavia) softwares. Unpaired two-tailed t-test was used to compare experimental groups. For comparison of TF and EphA2 expression in colorectal cancer non-parametric correlation was calculated according to Spearman, and Chi-squared tests were used to compare groups defined by TF and/or EphA2. *P*-values equal to or below 0.05 were considered statistically significant.

## Results

### Subcellular localization of TF and EphA2

**Fig. 1:**
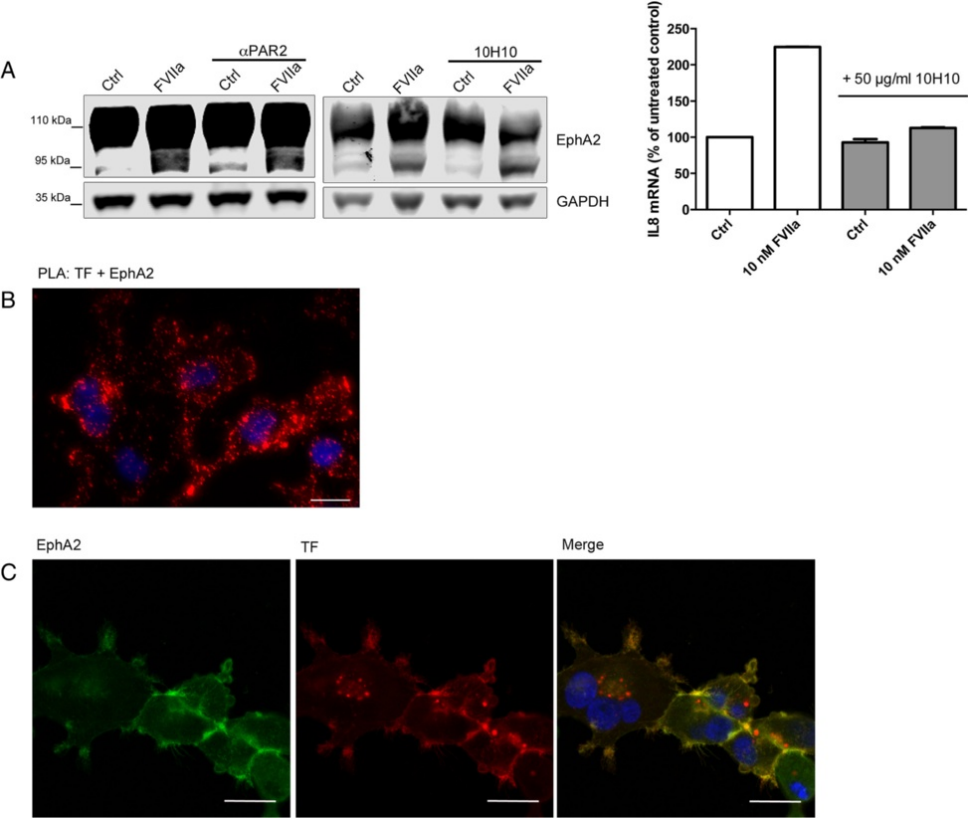
Co-localization between TF and EphA2 in MDA-MB-231 cells. **a** EphA2 cleavage is independent of PAR2. MDA-MB-231 cells were pretreated with 50 μg/ml anti-TF antibody 10H10 or 100 μg/ml PAR2-blocking antibody for 30 min followed by 10 nM FVIIa for 6 h. Images show representative Western blots. The 10H10 antibody was verified to prevent IL8 mRNA induction by FVIIa (graph). MDA-MB-231 cells were pre-treated with 50 μg/ml 10H10 antibody for 30 min, and 10 nM FVIIa was added for 1 h. FVIIa increased IL8 mRNA to 224.6 ± 0.3 %, whereas preincubation with mab10H10 abolished induction to 112.6 ± 1.2 %. *N* = 2, results are presented as percent of untreated control with error bars indicating the standard deviation. **b** EphA2 and TF in proximity were detected in MDA-MB-231 cells using an in situ proximity ligation assay with antibodies towards TF and EphA2. Red signal corresponds to TF-EphA2 complexes, blue signal represents DAPI-stained DNA. **c** Confocal micrographs showing MDA-MB-231 cells stained for EphA2 (green signal) and TF (red signal). Blue signal represents DAPI-stained DNA. Scale bar represents 20 μm

We recently identified EphB2 and EphA2 as novel proteolytical substrates of TF/FVIIa, and located the cleavage site to a conserved arginine residue in the ligand-binding domain [16]. Here we set out to further analyze the EphA2 cleavage mechanism. As seen in Fig. 1, EphA2 is cleaved upon stimulation of MDA-MB-231 breast cancer cells with FVIIa, with the truncated EphA2 isoform appearing as band migrating around 95 kDa on SDS-PAGE. In line with a direct cleavage mechanism, we previously showed that EphB2 was cleaved independently of proteolytic activation of the prototypic TF/FVIIa signaling receptor PAR2. Here we confirmed the PAR2-independence for EphA2 cleavage, as formation of the truncated EphA2 species by TF/FVIIa was not prevented by a PAR2-blocking antibody or the 10H10 anti-TF antibody specifically preventing PAR2-activation [21] (Fig. 1a), demonstrating a similar mechanism as for EphB2. The PAR2-blocking antibody was verified in our lab previously [22] and we also found that the 10H10 antibody prevented TF/FVIIa-PAR2 induced IL8 mRNA transcription as has been reported by others [21] (Fig. 1a).

**Fig. 2:**
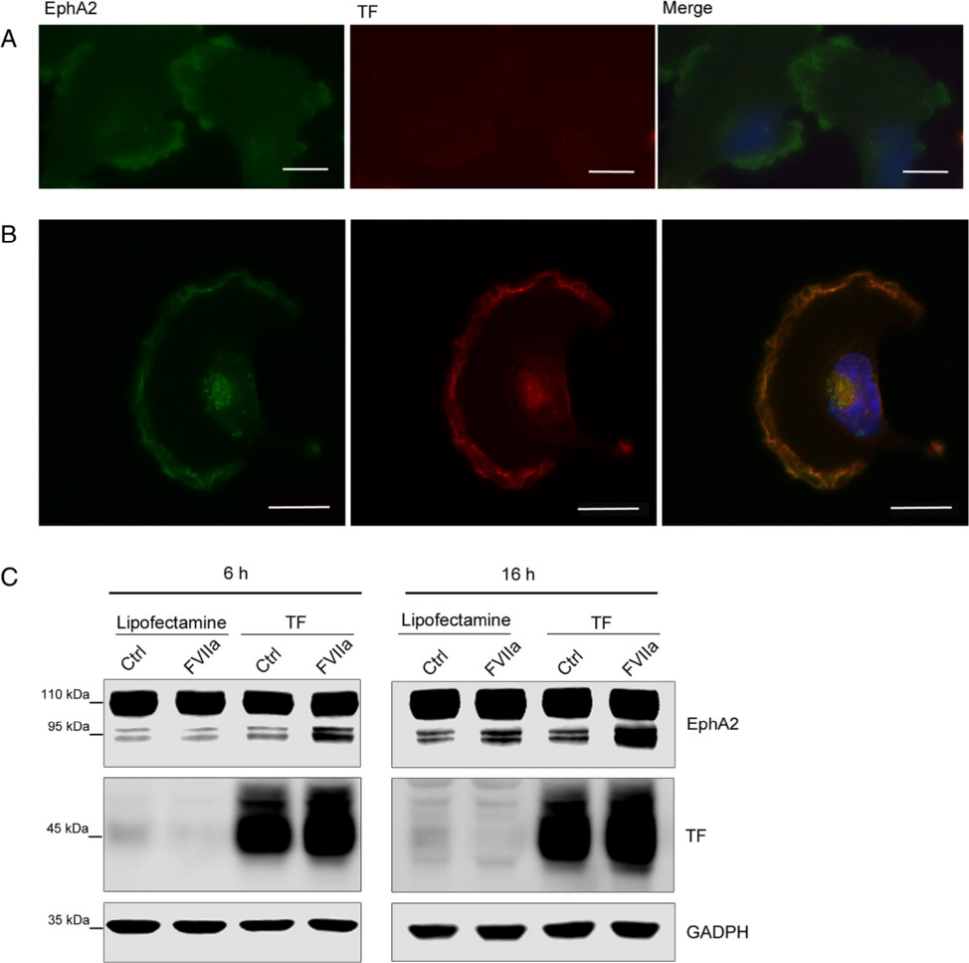
Overexpression of TF is required for EphA2 co-localization and cleavage in U251 cells. **a**-**b** U251 cells were transfected with a plasmid encoding human TF or treated with Lipofectamine alone as control. The figure shows confocal micrographs of control (**a**) or TF transfected (**b**) U251 cells stained for EphA2 (green signal) and TF (red signal). Blue signal represents DAPI-stained DNA. Scale bar represents 20 μm. **c** Lipofectamine-treated or TF-transfected U251 cells were treated with 10 nM FVIIa for the indicated time points. Samples were analyzed for EphA2 and TF expression by Western blot. *N* = 2–3, representative blots are shown

These results supported a direct cleavage mechanism, which would require a co-localization between the protease and the substrate. Using the in situ proximity ligation method [23] to detect protein interactions we found that TF and EphA2 constitutively co-localized in MDA-MB-231 cells as measured by an abundance of signals corresponding to TF and EphA2 in close proximity (Fig. 1b). We also immunostained MDA-MB-231 cells for TF and EphA2 and generated micrographs by confocal microscopy. TF and EphA2 both localized to cell membranes and co-localization was especially evident at cell-cell contacts (Fig. 1c), supporting an association between TF/FVIIa and EphA2. Secondary antibody controls showed no evidence for unspecific staining (Additional file 1). We compared these results to the U251 glioblastoma cell line, which expresses high levels of EphA2 but low amounts of TF, and confocal microscopy analysis showed a strong membranous EphA2 staining but a weak TF signal (Fig. 2a). Furthermore, EphA2 cleavage by FVIIa was not detected in these cells even after prolonged stimulations (Fig. 2c). We reasoned that a higher expression of TF than what was present in wild-type cells was necessary to allow sufficient complex formation with EphA2 at the cell surface, and in order to test this hypothesis we increased TF expression in these cells by transient overexpression. In contrast to wild type cells, TF appeared in transfected cells as an intense membranous staining with strong clustering at the leading edge and evident co-localization with EphA2 (Fig. 2b). In TF-transfected, but not wild-type cells, stimulation with 10 nM FVIIa resulted in robust EphA2 cleavage indicating that high levels of TF expression resulting in co-localization with EphA2 is necessary for FVIIa to be able to cleave EphA2 (Fig. 2c).

### FVIIa potentiates ephrin-A1 induced cell rounding independently of PAR2-cleavage

**Fig. 3:**
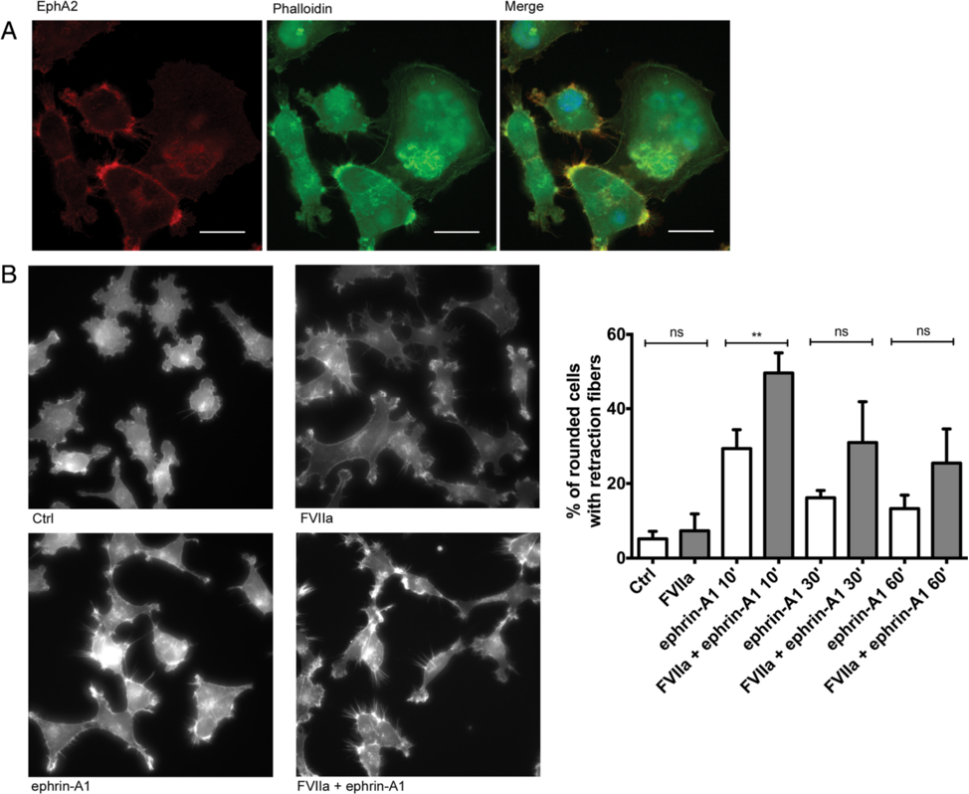
EphA2 co-localize with f-actin and FVIIa stimulation potentiates ephrin-A1-induced cell rounding and retraction fiber formation in MDA-MB-231 cells. **a** EphA2 co-localizes with the actin cytoskeleton in MDA-MB-231 cells. Confocal micrographs showing EphA2 (red signal) and actin fibers stained by phalloidin (green signal). Blue signal represents DAPI-stained DNA. **b** MDA-MB-231 cells were plated on collagen IV and treated with 10 nM FVIIa for 1 h prior to stimulation with 1 μg/ml ephrin-A1 for 10, 30 and 60 min. Cells were fixed and stained with FITC-conjugated phalloidin and micrographs generated by epifluorescence microscopy. Ephrin-A1 treatment induced cell rounding and retraction fiber formation, which was potentiated by FVIIa to 49.7 ± 4.4 % vs 29.3 ± 4.1 % at 10 min (*p* =0.009), 30.9 ± 8.9 % vs 16.2 ± 1.6 % at 30 min (*p* = 0.08) and 25.4 ± 7.5 % vs 13.3 ± 2.9 % at 60 min (*p* = 0.010). FVIIa alone did not increase cell rounding and retraction fiber formation compared to untreated cells (7.3 ± 4.5 % vs 5.2 ± 2 %, *p* = 0.49). *N* = 3, results are expressed as means ± SD of the percentage of rounded cells with retraction fibers. Representative images are shown

Given the interaction between TF and EphA2 we next set out to investigate how formation of the TF/FVIIa complex would affect EphA2 signaling and its activation by the ligand ephrin-A1. Ligand-dependent forward signaling by EphA2 frequently targets the cytoskeleton, with EphA2 activation by ephrin-A1 leading to cytoskeletal rearrangements and cell rounding [17]. We stained MDA-MB-231 cells with phalloidin to visualize the actin cytoskeleton and found that EphA2 was enriched at clusters of dynamic actin fibers and at cell-cell contacts (Fig. 3a).

As demonstrated by others [17, 24], stimulation with ephrin-A1 caused a transient increase in cell rounding which peaked at 10 min. Phalloidin staining revealed an accumulation of actin fibers at cell borders and an increase in retraction fibers, which were formed as cells rounded up and retracted their protrusions [25]. To investigate a role for TF/FVIIa in this process cells were pre-incubated with 10 nM FVIIa, and then simulated with ephrin-A1 followed by phalloidin staining. In this context FVIIa alone caused no detectable changes in cell morphology. However, upon FVIIa pretreatment the cellular response to ephrin-A1 was greatly enhanced. The fraction of rounded cells with retraction fibers upon ephrin-A1 stimulation was increased by FVIIa preincubation at all time points, demonstrating a potentiation of ligand-dependent EphA2 activation (Fig. 3b).

**Fig. 4:**
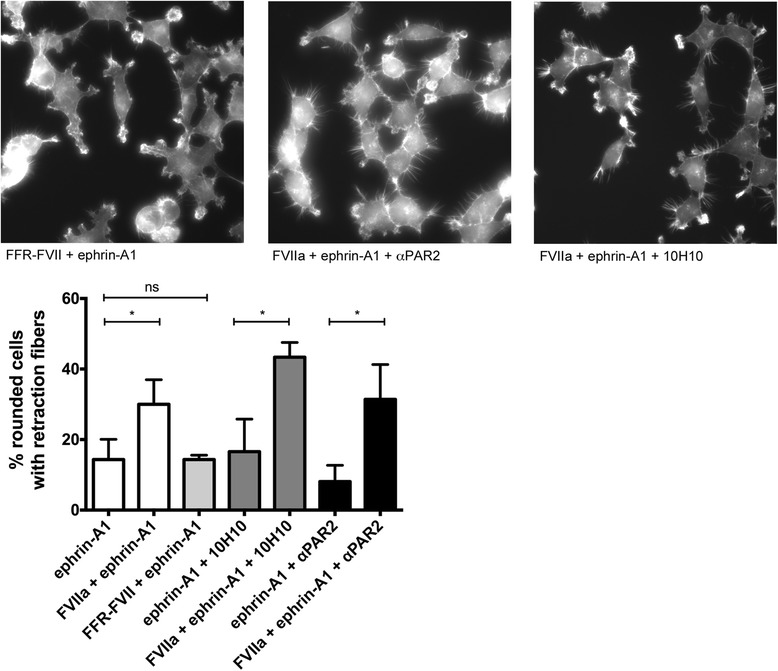
Potentiation of ephrin-A1-induced cell rounding and retraction fiber formation by FVIIa is independent of PAR2. MDA-MB-231 cells were pretreated with 50 μg/ml anti-TF antibody 10H10 or 100 μg/ml PAR2-blocking antibody for 30 min, stimulated with 10 nM FVIIa for 1 h and then 1 μg/ml ephrin-A1 for 10 min. Alternatively, 10 nM active-site inhibited FVII (FFR-FVII) was added instead of FVIIa. Potentiation of cell rounding and retraction fiber formation was not prevented by the anti TF 10H10 antibody (43.4 ± 4.2 % vs 16.6 ± 9.2 %, *p* = 0.01) or a PAR2-blocking antibody (31.4 ± 9.9 % vs 8.1 ± 4.6 %, *p* = 0.02), FFR-FVII did not increase cell rounding and retraction fiber formation together with ephrin-A1 (14.3 ± 5.7 % vs 14.4 ± 1.2 %, *p* = 0.99). *N* = 3, results are expressed as means ± SD of the percentage of rounded cells with retraction fibers. Representative images are shown

As FVIIa cleaved EphA2 independently of PAR2 we tested the requirement of PAR2 in this context. Blocking experiments with an anti-PAR2 antibody and the anti-TF 10H10 antibody which selectively prevents PAR2 activation did not interfere with the synergistic effects of FVIIa and ephrin-A1 on cell rounding, demonstrating that this occurs independently of PAR2 (Fig. 4). Experiments with active site-inhibited FVII (FFR-FVII) confirmed the requirement for the proteolytical activity of FVIIa to synergistically enhance the ephrin-A1 response (Fig. 4).

### Cell rounding induced by FVIIa and ephrin-A1 is dependent on a RhoA/ROCK pathway

**Fig. 5:**
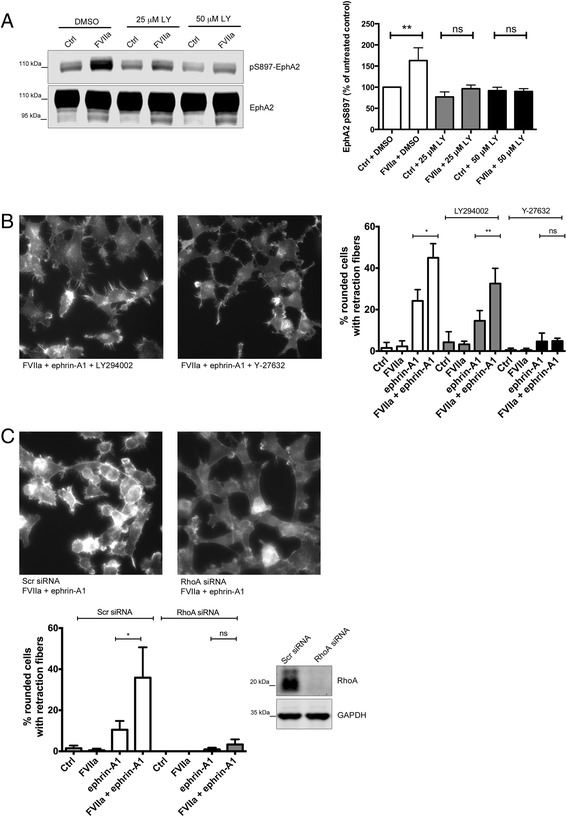
Cell rounding and retraction fiber formation in response to FVIIa and ephrin-A1 is dependent on a RhoA/ROCK pathway. **a** MDA-MB-231 cells were pre-incubated with either DMSO or 25 or 50 μM PI3K inhibitor LY294002 before 10 nM FVIIa was added for 1 h. Samples were analyzed by Western blot. S897-phosphorylation was increased to 163.1 ± 30.2 % of control in DMSO-treated cells (*p* = 0.006), whereas 25 μM or 50 μM LY294002 abolished induction by FVIIa to 96.4 ± 8.6 % vs 77.1 ± 11.7 % (*p* = 0.08) or 89.9 ± 6.7 % vs 91.7 ± 8.1 % (*p* = 0.78), respectively. Values are expressed as % of control. *N* = 3–4, results are presented as means ± SD. **b** MDA-MB-231 cells were pretreated with 10 μM ROCK-inhibitor Y-27632 or 25 μM PI3K inhibitor LY294002 for 30 min and then stimulated as in Fig. 4. Y-27632 pre-treatment abolished the effects of ephrin-A1 as well as the potentiation by FVIIa (4.8 ± 1.3 % vs 4.6 ± 4.1 %, *p* = 0.93), while LY294002 had no effect (32.5 ± 7.3 % vs 14.6 ± 4.9 %, *p* = 0.007). *N* = 3–4, results are expressed as means ± SD of the percentage of rounded cells with retraction fibers. **c** RhoA expression was silenced by siRNA in MDA-MB-231 cells, and knock-down was verified by Western blot. In Scr-transfected cells FVIIa potentiated ephrin-A1 induced cell rounding (35.87 ± 14.80 vs 10.49 ± 4.29 %, *p* = 0.046), whereas RhoA knock-down cells were unresponsive to ephrin-A1 and FVIIa (3.30 ± 2.50 % vs 0.92 ± 0.88 %, *p* = 0.19). *N* = 3, results are expressed as means ± SD of the percentage of rounded cells with retraction fibers

We next sought to gain insight into the downstream signaling mechanisms. A serine residue in the cytoplasmic domain of EphA2, serine 897 (S897), was recently discovered as an important phosphorylation site mediating cell motility downstream of PI3K/Akt signaling [9]. Western blots of FVIIa-treated MDA-MB-231 cells revealed a strong increase in S897 phosphorylation by 10 nM FVIIa, which was completely abolished by the PI3 kinase inhibitor LY294002 (Fig. 5a). In contrast, EphA2 cleavage was not affected by PI3 kinase inhibition (Fig. 5a), in agreement with a direct cleavage by FVIIa and demonstrating that TF/FVIIa targets EphA2 by two independent mechanisms. We tested the PI3K-dependence in the cell rounding assay, but the PI3K inhibitor LY294002 did not affect the synergistic effect of FVIIa and ephrin-A1, ruling out a mechanism dependent on this pathway (Fig. 5b).

Instead, ligand-dependent EphA2 activity targeting the cytoskeleton has been reported to be dependent on RhoA signaling [26]. Preincubation of cells with 10 μM Y-27632, an inhibitor of the RhoA signaling effector ROCK, abolished cell rounding by ephrin-A1 and FVIIa (Fig. 5b). We confirmed this result by knocking down RhoA with siRNA. RhoA-depleted cells were almost completely unresponsive to ephrin-A1 with regards to cytoskeletal rearrangements and maintained a spread morphology both when treated with ephrin-A1 alone or in combination with FVIIa, showing a mechanism dependent on a RhoA/ROCK pathway downstream of EphA2 (Fig. 5c).

**Fig. 6:**
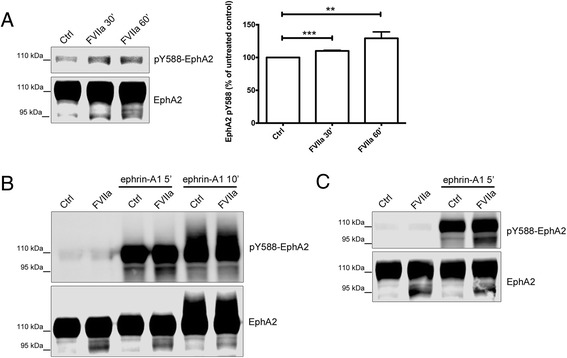
EphA2 is tyrosine phosphorylated by FVIIa and ephrin-A1. **a** MDA-MB-231 cells were treated with 10 nM FVIIa and samples were analyzed by Western blot for EphA2 phosphorylation on tyrosine 588 (Y588) and total EphA2 protein. FVIIa increased the phosphotyrosine signal to 110.0 ± 1.02 % (*p* < 0.001) after 30 min and 129.2 ± 9.82 % (*p* = 0.0067) after 60 min. *N* = 3, results are presented as means ± SD. **b** MDA-MB-231 cells were pretreated with 10 nM FVIIa for 1 h and then stimulated with 1 μg/ml ephrin-A1-Fc for 5 or 10 min. **c** MDA-MB-231 cells were pretreated with 10 nM FVIIa for 6 h and then stimulated with 1 μg/ml ephrin-A1-Fc for 5 min. Samples were analyzed by Western blot as in (**a**). *N* = 2–3, representative gels are shown

These results demonstrated a synergistic effect of ephrin-A1 and FVIIa in EphA2 activation independent of PAR2-signaling and PI3K-dependent serine phosphorylation of EphA2, and EphA2 was cleaved by TF/FVIIa in a PAR2- and PI3K-independent manner. Yet, the cleavage by TF/FVIIa occurs in the EphA2 LBD. However, the N-terminal fragment remains attached to the truncated EphA2 species by a disulfide bond [16], raising the possibility that it might still be responsive to ephrin-A1. Being an RTK, EphA2 has intrinsic tyrosine kinase activity that is activated by ligand-binding and we examined EphA2 activation by FVIIa and ephrin-A1 by using an antibody towards phosphorylated tyrosine 588 (Y588) in the EphA2 cytoplasmic domain. Western blots showed low tyrosine phosphorylation in the basal state, which was slightly but consistently increased by FVIIa as seen on high-exposure Western blots (Fig. 6a). Ephrin-A1 treatment resulted in a rapid and strong tyrosine phosphorylation of full-length EphA2 that, however, was not further increased by FVIIa pretreatment. The cleaved EphA2 species was also rapidly tyrosine phosphorylated by ephrin-A1, which was detected after 1 h or 6 h pre-treatment with FVIIa (Fig. 6b-c). Cleaved EphA2 also appeared to undergo rapid ligand-induced downregulation, as we consistently found a time-dependent reduction of the band corresponding to cleaved EphA2 after ephrin-A1 stimulation, as seen on the 10 min ephrin-A1 time point in Fig. 6b. Taken together, these results indicate that cleaved EphA2 is activated in response to stimulation with ephrin-A1.

### TF and EphA2 are co-expressed in advanced stage colorectal cancers and appear close to necrotic and invasive areas

**Fig. 7:**
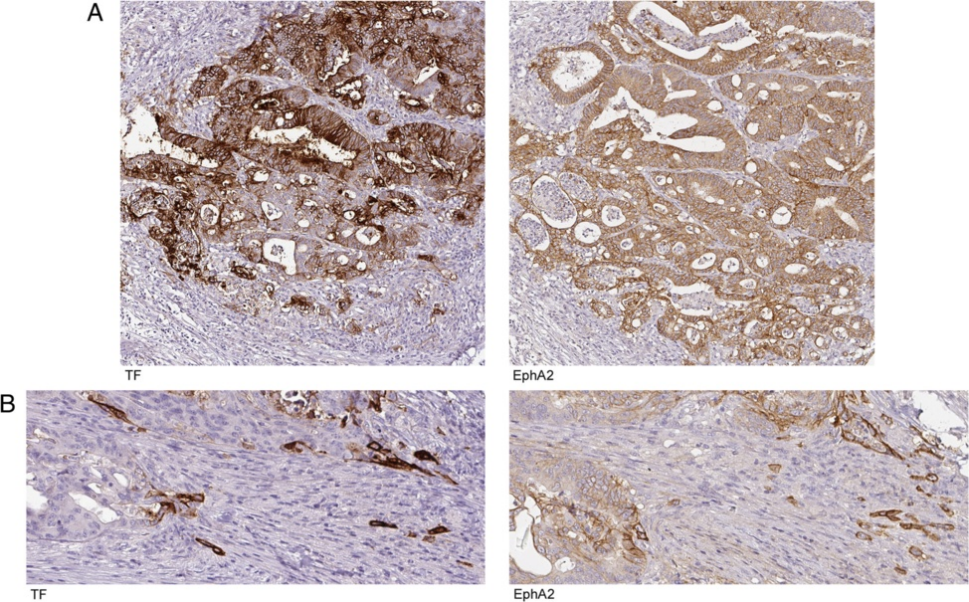
EphA2 and TF are co-expressed in a colorectal cancer. Representative images of immunohistochemistry stainings for TF and EphA2. Brown color represents positive staining. **a** Serial sections from specimen with high expression of TF (*left panel*) and EphA2 (*right panel*). Original magnification 20×. **b** Serial sections from specimen with scattered positivity for TF (*left panel*) and EphA2 (*right panel*) localized to necrotic areas and budding tumor cells. Original magnification 40 ×

TF expression has previously been detected in many solid tumors, including colorectal cancers [27], and has been linked to tumor progression on an experimental level using animal models and in vitro cell culture systems [13]. To explore if a role for TF-EphA2 cross-talk in this context is plausible we stained a cohort of colorectal cancer specimens for TF and EphA2 by IHC and scored their expression levels in tumor cells. IHC stainings on normal colorectal mucosa showed that epithelial cells in the large intestine were mostly negative for TF, while strong staining was seen in the epithelial lining. EphA2 expression was present in the most apical parts of the colorectal epithelium while basal portions of the glands were negative, in agreement with previous observations [28] and demonstrating antibody specificity (Additional file 1). EphA2 expression then reappeared in cancer specimens, along with increased positivity for TF. TF expression was detected in 28 % of primary cancers and 29 % of lymph gland or distant metastases, and EphA2 in 59 % of cases in both groups (Table 1). Interestingly, annotation scores for TF and EphA2 expression correlated (Spearman Rho 0.48, *p* < 0.001), with 87 % of TF positive primary tumors and 100 % of TF positive metastases also being positive for EphA2, demonstrating that TF and EphA2 are co-expressed in colorectal carcinomas. We also investigated if combined expression of TF and EphA2 was related to tumor characteristics in primary tumors. Co-expression of TF and EphA2, compared to negativity for either of the two proteins, was significantly associated with poorly differentiated histology (high grade tumors), as six out of 13 (46 %) cases in this group were double positive compared to only seven out of 39 (18 %) cases with intermediate/high differentiation (low to intermediate grade) (*p* = 0.042) (Table 2). Notably, staining of serial sections revealed that TF and EphA2 positivity appeared to be concentrated to clusters of tumor cells close to necrotic areas or small clusters of budding tumor cells invading through the stroma (Fig. 7a–b).

**Table 1:** Association between TF and EPHA2 expression in colorectal cancer

	Total *N* (%)	TF positive *N* (%)	TF negative *N* (%)	*P* value
Primary cancers				
All cases	54	15	39	n/a
EphA2				
positive	32 (59)	13 (87)	19 (49)	**0.011**
negative	22 (41)	2 (13)	20 (51)	
Metastases				
All cases	17	5	12	n/a
EphA2				
positive	10 (59)	5 (100)	5 (42)	**0.026**
negative	7 (41)	0 (0)	7 (58)	

*P*-values in bold indicate statistically significant results

**Table 2:** Associations between TF and EPHA2 positive colorectal cancers and patient and tumor characteristics

Characteristic	Total N (%)	TF & EphA2 pos N (%)	TF or EphA2 neg N (%)	*P* value
All cases	54	13	41	n/a
Stage				
Stage I Stage II Stage III	20 (37) 19 (35) 15 (28)	5 (38.5) 3 (23) 5 (38.5)	15 (37) 16 (39) 10 (24)	0.49
Grade				
Low/Intermediate High (Low diff) Missing	39 (75) 13 (25) 2	7 (54) 6 (46)	32 (82) 7 (18)	**0.042**
Location				
Colon Rectum	37 (69) 17 (31)	10 (77) 3 (23)	27 (66) 14 (34)	0.45
Sex				
Male Female	23 (43) 31 (57)	7 (54) 6 (46)	16 (39) 25 (61)	0.35
Ki67				
>25 % <25 %	43 (80) 11 (20)	11 (85) 2 (15)	32 (78) 9 (22)	0.61
CK20				
Positive Negative	49 (91) 5 (9)	12 (92) 1 (8)	37 (90) 4 (10)	0.82

*P*-values in bold indicate statistically significant results

## Discussion

We report herein on a close cross-talk between TF and the tyrosine kinase receptor EphA2 and present evidence of a role for the TF/FVIIa complex as a co-receptor and signaling partner of EphA2 with possible implications in human cancer. We observed that TF and EphA2 co-localized in MDA-MB-231 breast cancer cells with high endogenous TF expression, and in U251 glioblastoma cells with forced overexpression of TF. EphA2 and TF appeared to cluster at cell-cell contacts and subcellular compartments with an accumulation of dynamic actin cytoskeleton, in agreement with literature documenting an important role for EphA2 in regulating cytoskeletal dynamics [29, 30]. Importantly, we found that FVIIa potentiated the cellular response to ephrin-A1 as measured by increased cell rounding and retraction fiber formation upon stimulation, demonstrating that FVIIa and ephrin-A1 act synergistically to enhance ligand-dependent EphA2 signaling. By antibody blocking experiments, we show that this is an event uncoupled from PAR2-activation, in line with biochemical data demonstrating direct cleavage of EphA2 by TF/FVIIa, and supporting a role of the TF/FVIIa complex acting as a co-receptor in EphA2 signaling.

EphA2 is cleaved by FVIIa after a conserved arginine residue in the J-K loop of the LBD, and we previously showed that the cleaved fragment remains associated to the truncated EphA2 by a conserved disulfide bond (Cys70-Cys188), and the LBD is also stabilized by an additional disulfide (Cys105-Cys115). Since the Cys70-Cys188 disulfide will prevent dissociation of the N-terminal fragment we predict that the structure of the EphA2 LBD is largely retained after cleavage, with the cleavage leading to a local conformational change in the J-K loop. We hypothesize that cleavage by TF/FVIIa might, by a yet unidentified exact mechanism, enhance EphA2 activation by its ligand. As it was tyrosine phosphorylated and rapidly underwent ligand-induced degradation, our data indicate that the cleaved fragment indeed contributes to ephrin-A1-dependent signaling and that the cleavage does not results in a ligand-unresponsive form of EphA2. Of note, as the synergism between FVIIa and ephrin-A1 was PAR2-independent in line with the cleavage mechanism, it appears not to be an unrelated event resulting from PAR2 activation by TF/FVIIa.

EphA2 tyrosine phosphorylation was very low in unstimulated cells, which was expected since MDA-MB-231 cells are reported to express very low amounts of the ephrin-A1 ligand [8]. We observed a slight increase of phosphorylation at the Y588 site by FVIIa, but since this effect was negligible compared to the response induced by ephrin-A1 the relevance of this observation with regards to synergism between FVIIa and ephrin-A1 is not clear at the moment. Of note, we previously analyzed this cell line using antibody arrays [16], where FVIIa decreased the phosphotyrosine signal for EphA2 and as we noted, since those experiments were run with native samples a decrease in signal can equally well correspond to masking of the phosphotyrosine epitope by proteins recruited to the activated receptor [31].

Even though a large number of studies have confirmed an important role of EphA2 in human cancers, there are controversies regarding the contributions of ligand dependent and ligand independent signaling. Ephrin-A1 ligation of EphA2 and subsequent receptor activation has been proposed to be tumor suppressive, although other studies point to an important role for kinase dependent EphA2 activity in cancer development. Cell rounding and retraction is a recognized event in Eph-ephrin signaling, and requires RhoA activation [6, 7], in accordance with our results. Interestingly, recent work suggest that EphA2-ephrin-A1 signaling may support cell migration and invasion in prostate cancer [5] and melanoma cells [6] in this manner. Here, RhoA-mediated mesenchymal-to-amoeboid transition has been implicated, indicating a qualitative shift in cell migration characterized by cell rounding, single cell invasion and a squeezing movement style, leading to tissue invasion and tumor dissemination [32, 33]. Also, other studies demonstrated that repulsive EphA2-RhoA signaling among cancer cells may serve to allow tumor cells to disperse from the main tumor mass [26]. Importantly, contact inhibition of locomotion which may occur as a consequence of repulsive interactions between Eph and ephrin expressing cells does not need to lead to a complete inhibition of motility but rather a change in direction, in certain contexts contributing to a shift from collective to single cell cancer invasion [34]. In this context, our results demonstrating a synergism of FVIIa and ephrin-A1 in EphA2 activation lead us to hypothesize that TF/FVIIa may serve to increase EphA2-mediated cell dispersion upon contact with ephrin-A1 expressing cells, and that FVIIa may facilitate ephrin-A1 induced mesenchymal-to-amoeboid transition in this context.

EphA2 signaling is characterized by a high level of complexity and several seemingly independent activation mechanisms, as demonstrated by the present study where FVIIa caused both a direct cleavage and S897 phosphorylation of EphA2. While cleavage was insensitive to PI3 kinase inhibition, S897 phosphorylation was completely abolished in agreement with the notion that this is a common event downstream of activation of PI3 kinase. Cell rounding and retraction fiber formation was not affected by PI3K inhibition, showing a dissociation between EphA2-pS897 signaling and ligand-dependent EphA2 activation as has been proposed elsewhere [9]. The role and function of FVIIa-induced EphA2 S897-phosphorylation was not elucidated in this study, and its role as an effector downstream of FVIIa-induced PI3K activation will be an important issue for further studies.

In support of the relevance of TF/FVIIa-EphA2 cross-talk we present descriptive data that TF and EphA2 were co-expressed to a high extent in a human cancer material, and TF positivity was with a few exceptions only found in EphA2 expressing tumors. TF and EphA2 have separately been reported to be expressed in various cancers, but our study is the first to demonstrate co-expression in the same tumor specimens. In contrast to our experimental work that was performed in breast cancer and glioblastoma cell lines, we used another cancer type, colorectal cancer, to demonstrate the association between TF and EphA2 in human tumor tissue. Although these are distinct cancer types, both TF and EphA2 expression appears to foremost be a feature of advanced cancers, and TF expression is regulated by common oncogenic mutations [35]. Indeed, EphA2 has been linked to colorectal cancer development as targeted EphA2 gene disruption was found to reduce intestinal tumorigenesis in the APC/Min + mouse model [36]. We found that co-expressing tumors were enriched among high-grade cases, supporting the view that these results may be a feature of poorly differentiated human cancers rather than a tissue specific event in colorectal neoplasms. TF expression was, for both primary cancers and metastases, found in a minority of cases, and our results indicate that TF and EphA2 co-expression is an event that occurs in a subset of colorectal cancers that are characterized by low differentiation. As a large number of cases were TF negative, this might be a mechanism that contributes to tumorigenesis in a subset of tumors, whereas other oncogenic pathways drive more differentiated cancers. Here, future studies on larger clinical materials will have to provide answers on the possible impact on patient survival and outcome.

We previously noted that higher concentrations of FVIIa were needed for cleavage of EphA2 compared to cleavage of another Eph receptor, EphB2. In the present study, overexpression of TF was required to achieve EphA2 cleavage by FVIIa in U251 cells and it thus appears that very high levels of TF, such as may be found in transformed cells are necessary for this to occur. However, high levels of both TF and EphA2 are recognized events in malignant cells, and we used a colorectal cancer material to show that co-expression of TF and EphA2 indeed occurs in vivo. In addition, cleavage of cellular subpopulations with EphA2 co-localizing with TF may also play important biological roles even though these are events that are below the detection limit of Western blot assays.

## Conclusion

In summary, we show with a proximity ligation assay and immunofluorescence that TF and EphA2 co-localize in cancer cell lines and that TF/FVIIa cleaves EphA2 in cells with high TF expression in a PAR2-independent manner. FVIIa stimulation increased ephrinA1-induced cell rounding mediated by a RhoA/ROCK pathway and thus potentiated ephrin-A1-EphA2 cytoskeletal rearrangements, with a potential role in mesenchymal-to-amoeboid transition mediated by the EphA2-ephrin-A1 axis. We also present initial evidence that EphA2 and TF are co-expressed in vivo in poorly differentiated colorectal tumors, thus warranting further studies on their cooperative role in human cancers.

## Abbreviations

FFR-FVII, active site-inhibited FVII; FVII/FVIIa, coagulation factor VII/VIIa; IHC, immunohistochemistry; LBD, ligand-binding domain; PAR2, protease-activated receptor 2; RTK, receptor tyrosine kinase; TF, Tissue Factor; TMA, tissue microarray.

## Additional file

Additional file 1: Supplemental Figures. (PDF 2.19 MB)
