# Supplementary material for: Cross-talk between the Tissue Factor/coagulation factor VIIa complex and the tyrosine kinase receptor EphA2 in cancer
Source: BMC Cancer. 2016 May 31;16:341. doi: 10.1186/s12885-016-2375-1 (PMC4888641; doi:10.1186/s12885-016-2375-1)
Supplement: Additional file 1: — Supplemental Figures. (PDF 2.19 MB) [file 12885_2016_2375_MOESM1_ESM.pdf]

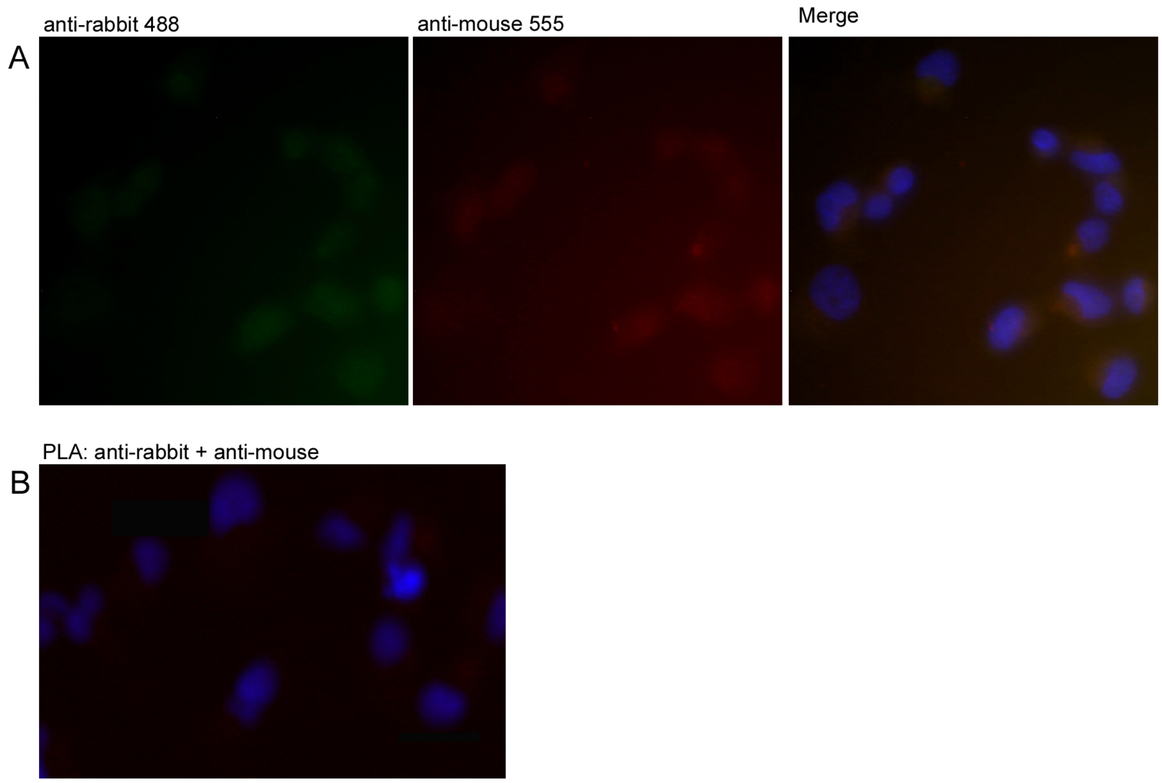

Supplemental Figure 1. Immunofluorescence secondary antibody controls.

(A) MDA-MB-231 cells were stained with secondary antibodies anti-rabbit^{488} (green channel) and anti-mouse^{555} (red channel). Blue represents DAPI stained DNA. Images were captured using a Zeiss epifluorescence microscope and the 40x objective.

(B) The PLA assay was performed on MDA-MB-231 cells omitting primary antibodies. Blue represents DAPI-stained DNA.

Images were captured using a Zeiss AxioVision Imager and the 40x objective.

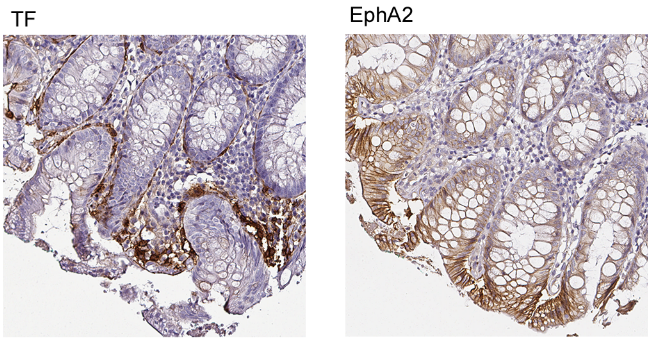

Supplemental Figure 2. Expression of TF and EphA2 in normal colorectal mucosa.

Serial sections of normal colorectal mucosa were stained for TF and EphA2 using IHC as described in Materials and Methods.

Positive staining is visible as brown color. Original magnification 20X.

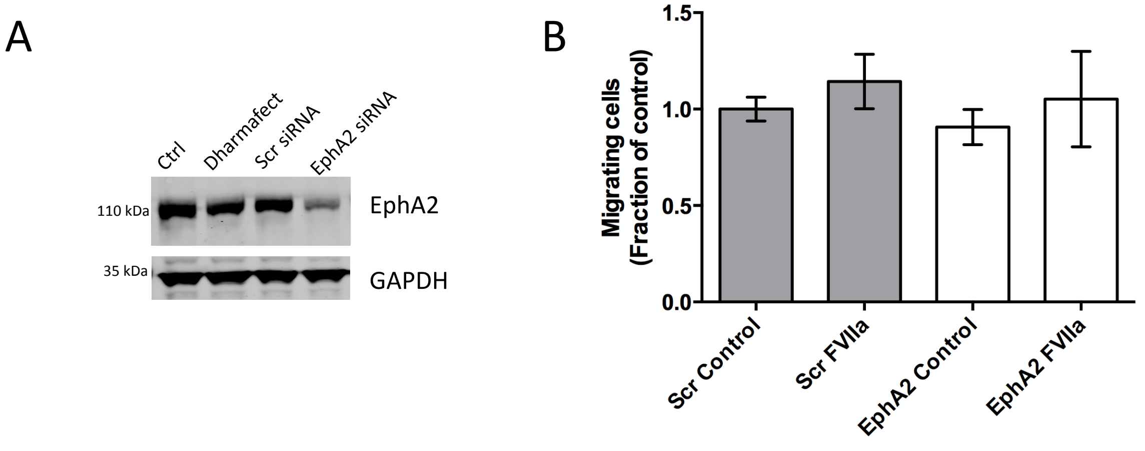

Supplemental Figure 3. EphA2 siRNA knockdown in MDA-MB-231 cells.

MDA-MB-231 cells were transfected with 10 nM scrambled or EphA2 siRNA (Silencer select, Ambion) using Dharmafect transfection reagent (Dharmacon) according to instructions from the manufacturer. Cells were re-transfected after 48 hours and assayed 72 hours after the initial transfection.

(A) Efficiency of protein knock-down was analyzed by Western blot.

(B) EphA2-transfected and Scr-transfected cells were subjected to a Transwell cell migration assay performed as previously described (Eriksson et al, Journal of Biological Chemistry 2014) with some modifications. Cells were resuspended in serum-free media (SFM) and allowed to migrate towards SFM supplemented with 10 nM FVIIa. After 5 h, the inserts were removed, fixed and stained with Hoechst stain, and migrating cells on the underside of the inserts were counted with the Cell Profiler software (www.cellprofiler.org).
